# Naturally occurring a loss of a giant plasmid from *Mycobacterium ulcerans* subsp. *shinshuense* makes it non-pathogenic

**DOI:** 10.1038/s41598-018-26425-1

**Published:** 2018-05-29

**Authors:** Kazue Nakanaga, Yoshitoshi Ogura, Atsushi Toyoda, Mitsunori Yoshida, Hanako Fukano, Nagatoshi Fujiwara, Yuji Miyamoto, Noboru Nakata, Yuko Kazumi, Shinji Maeda, Tadasuke Ooka, Masamichi Goto, Kazunari Tanigawa, Satoshi Mitarai, Koichi Suzuki, Norihisa Ishii, Manabu Ato, Tetsuya Hayashi, Yoshihiko Hoshino

**Affiliations:** 1 Present Address: Department of Mycobacteriology, Leprosy Research Center, National Institute of Infectious Diseases, Tokyo, Japan; 2 Antimicrobial Resistance Research Center, National Institute of Infectious Diseases, Tokyo, Japan; 3 Department of Bacteriology, Faculty of Medicine, Kyushu University, Fukuoka, Japan; 4 Center for Information Biology, National Institute of Genetics, Shizuoka, Japan; 5 Department of Food and Nutrition, Faculty of Contemporary Human Life Science, Tezukayama University, Nara, Japan; 6 The Research Institute of Tuberculosis, Japan Anti-Tuberculosis Association, Tokyo, Japan; 7 Department of Microbiology, Graduate School of Medical and Dental Sciences, Kagoshima University, Kagoshima, Japan; 8 National Sanatorium Hoshizuka-Keiaien, Kagoshima, Japan; 9 Present Address: School of Pharmacy, Hokkaido Pharmaceutical University, Sapporo, Japan; 10 Faculty of Pharma-Sciences, Teikyo University, Tokyo, Japan; 11 Department of Clinical Laboratory Science, Faculty of Medical Technology, Teikyo University, Tokyo, Japan

## Abstract

*Mycobacterium ulcerans* is the causative agent of Buruli ulcer (BU), a WHO-defined neglected tropical disease. All Japanese BU causative isolates have shown distinct differences from the prototype and are categorized as *M. ulcerans* subspecies *shinshuense*. During repeated sub-culture, we found that some *M. shinshuense* colonies were non-pigmented whereas others were pigmented. Whole genome sequence analysis revealed that non-pigmented colonies did not harbor a giant plasmid, which encodes elements needed for mycolactone toxin biosynthesis. Moreover, mycolactone was not detected in sterile filtrates of non-pigmented colonies. Mice inoculated with suspensions of pigmented colonies died within 5 weeks whereas those infected with suspensions of non-pigmented colonies had significantly prolonged survival (>8 weeks). This study suggests that mycolactone is a critical *M. shinshuense* virulence factor and that the lack of a mycolactone-producing giant plasmid makes the strain non-pathogenic. We made an avirulent mycolactone-deletion mutant strain directly from the virulent original.

## Introduction

Buruli ulcer (BU) is one of twenty neglected tropical diseases (NTD) as defined by the World Health Organization (WHO)^1^. After leprosy and tuberculosis, BU is now the third most common mycobacterial disease worldwide^1^. BU is characterized as a chronic, indolent, necrotizing disease of skin and soft tissue that manifests as massive ulcers that result in permanent, disabling scars^2^.

The first reported case of BU in Japan occurred in a young woman who had never traveled abroad^3,4^. Based on differences in growth and biochemical properties from prototypic *M. ulcerans*, the causative agent for this patient’s BU was determined to be a *M. ulcerans* subspecies, *M. ulcerans* subsp. *shinshuense* (hereafter “*M. shinshuense*”)^3–5^. The growth rate of “*M. shinshuense*” was more rapid than that of *M. ulcerans* and “*M. shinshuense*” was positive for urease activity^3^. The incidence of “*M. shinshuense*” in Japan has gradually increased; as of December 2017, a total of 67 cases have been reported and 35 clinical strains have been isolated in our laboratory^6,7^.

Although “*M. shinshuense*” is considered to be an *M. ulcerans* subspecies, a recent whole genome sequence (WGS) analysis, which included a giant plasmid from the reference strain, revealed significant genetic differences between them^8^. Nonetheless, both *M. ulcerans* and “*M. shinshuense”* carry the giant plasmid (pMUM001) that harbors genes encoding the polyketide synthases required for mycolactone synthesis^9,10^. The macrolide mycolactone is a lipid-like exotoxin that has a structure similar to immune-suppressive agents such as cyclosporine, rapamycin, and tacrolimus^11,12^. Production of this toxin is critical for *M. ulcerans* pathogenesis.

We have noticed pigmented and non-pigmented colonies in the culture of “*M. shinshuense”*, which led us to explore the possible difference of the nature of these two phenotypes.

## Results

### Biochemical characteristics

**Figure 1:**
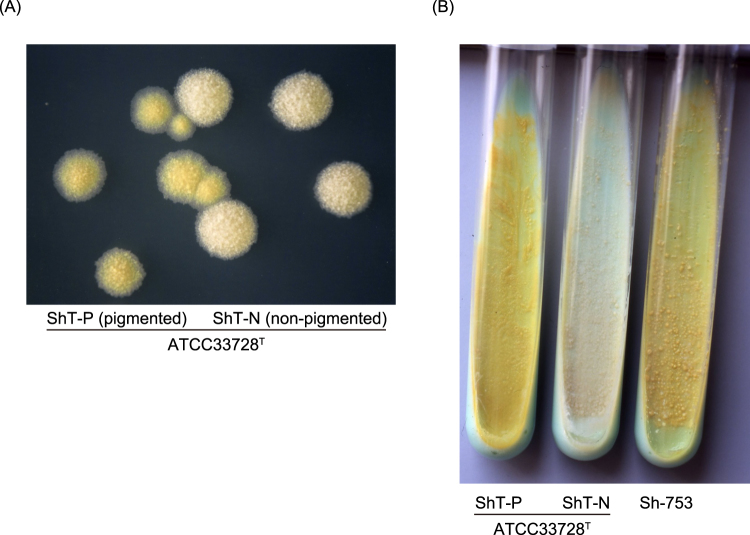
Characteristics of ShT-N and ShT-P colonies. (**A**) A mixture of pigmented (ShT-P) and non-pigmented (ShT-N) colonies of ATCC 33728^T^ on 7H11 solid medium. (**B**) Subcloned colonies of ATCC 33728^T^. ShT-P and ShT-N were isolated and propagated. Only pigmented, yellow colonies were produced by ShT-P, whereas for ShT-N only non-pigmented, ivory colonies reproduced. The mycolactone-producing “*M. shinshuense*” strain Sh-753 was used as a positive control.

We found ivory (non-pigmented) colonies among yellow (pigmented) “*M. shinshuense”* reference strain ATCC 33728 colonies during routine culture work (Fig. 1A). Each type of colony (labeled as ShT-P (pigmented) or ShT-N (non-pigmented)) was sub-cloned and purified by continuous three generation passage on 2% Ogawa egg medium slants (Fig. 1B).

Both ShT-P and ShT-N formed rough colonies that were visible within 14 days of inoculation. ShT-P retained the yellow pigment (and even when glowed in the dark), whereas ShT-N was not pigmented. Both formed visible colonies at 25 °C and 32 °C, but not at 37 °C or 42 °C. Neither grew on a medium supplemented with 500 μg/ml *p*-nitrobenzoic acid or 5% NaCl. Both isolates were negative for Tween 80 hydrolysis, nitrate reduction, arylsulfatase (3 days), pyrazinamidase, iron uptake, and MPB64 production, but were positive for semiquantitative catalase, 68 °C catalase and urease activities. The biochemical and growth characteristics of both “*M. shinshuense”* isolates, a different clinical isolate of “*M. shinshuense”* (Sh-753), and several *M. ulcerans* strains are summarized in Table 1. The results were in accordance with those of a previous report on *M. ulcerans*, except for the positive urease activity of the “*M. shinshuense”* isolates^2^.

**Table 1:** Characteristics of bacteria tested in this study.

	*“M. shinshuense”*			*M. ulcerans*	
	ATCC 33728			MU-4	MU-8
	ShT-P (pigmented)	ShT-N (non-pigmented)	Sh-753		
Mycolactone production	+	−	+	+	−
Colony:					
Color (pigmentation)	+	−	+	+	−
Morphology	R	R	R	R	R
Growth on 2% Ogawa medium slant					
14 days	+	+	+	−	−
30 days	+	+	+	+	+
Growth at:					
25 °C	+	+	+	+	+
32 °C	+	+	+	+	+
37 °C	−	−	−	−	−
42 °C	−	−	−	−	−
Growth on 500 μg/ml *p*-nitrobenzoic acid	−	−	−	−	−
Growth on NaCl medium (5%)	−	−	−	−	−
Tween 80 hydrolysis	−	−	−	−	−
Nitrate reduction	−	−	−	−	−
Arylsulfatase	−	−	−	−	−
Pyrazinamidase	−	−	−	−	−
Iron uptake	−	−	−	−	−
MPB64 Ag production	−	−	−	−	−
Semi−quantitative catalase	+	+	+	+	+
68 °C catalase	+	+	+	+	+
Urease	+	+	+	−	−

**Figure 2:**
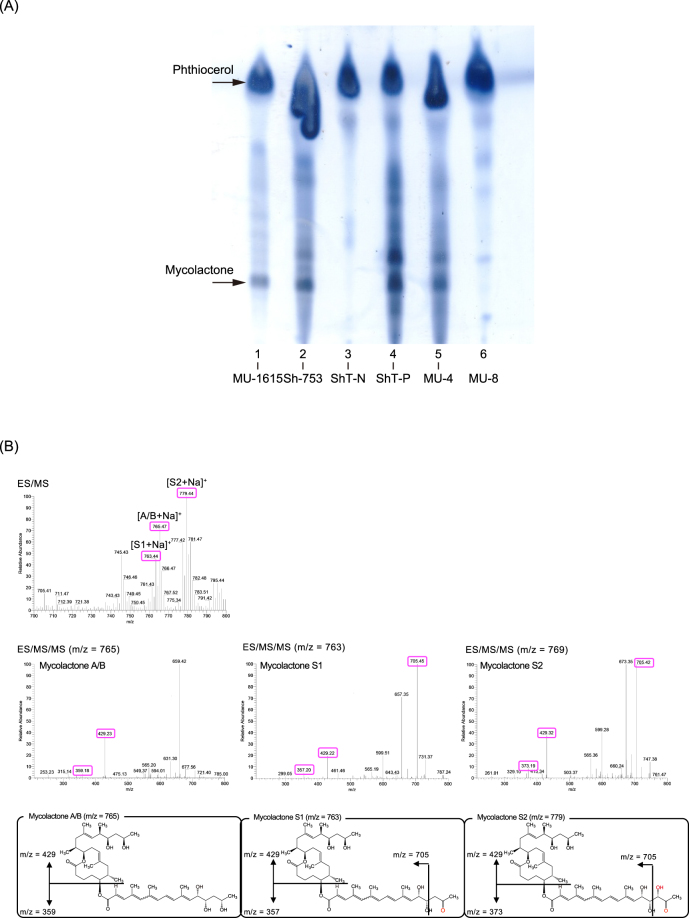
Silica thin-layer chromatography and ESI/MS/MS analyses of acetone soluble lipids (ASLs) from *M. ulcerans* or “*M. shinshuense*” isolates. (**A**) ASLs extracted from the bacterial cell mass (CM). Samples were loaded as follows: lane 1, MU-1615 (*M. ulcerans* Malaysian strain, virulent); lane 2, Sh-753 (“*M. shinshuense”*, virulent); lane 3, ShT-N (ATCC 33728, non-pigmented colony); lane 4, ShT-P (ATCC 33728, pigmented colony); lane 5, MU-4 (*M. ulcerans* 97–107 African strain, virulent); lane 6, MU-8; (*M. ulcerans* 5143 Mexican strain, avirulent). Arrows show the position of mycolactone and phthiocerol (control lipid). (**B**) The ESI/MS spectrum of ASLs from the strain ShT-P detected the peaks of m/z 765.4, 763.4, 779.4 as [M + Na]^+^. In each MS/MS spectrum, the distinct ions were found m/z 359 and 429 for mycolactone A/B; m/z 357, 429 and 705 for mycolactone S1; m/z 373, 429 and 705 for mycolactone S2, respectively. These molecular and fragment ions were fixed the proposed structures of each mycolactone.

R: rough. See Fig. 2 for visualization of mycolactone production by TLC.

### Genetic characteristics of *M. shinshuense* chromosomes

All multi-locus sequence typing (MLST) showed identical sequences between ShT-P and ShT-N. The 1,475–base pair (bp) 16S rRNA gene sequences of ShT-P and ShT-N were also identical, but both partially differed from those of related species such as *M. ulcerans*, *M. marinum*, and *M. pseudoshottsii* (Supplemental Table 1). The internal transcribed spacer (ITS) (272-bp), *rpoB* (315-bp), and *hsp65* (401-bp) sequences of ShT-P and ShT-N were also identical (data not shown).

A direct comparison of WGS data for ShT-P and ShT-N showed that the chromosomes of both isolates were almost identical except for 8 single nucleotide polymorphisms (SNPs) and 20 small insertions or deletions (Indels). Four discordant SNPs located in coding regions or intergenic regions, all of which could be aligned with WGS data and validated by Sanger sequencing. There was a non-synonymous mutation in three coding regions, whereas the other one SNP was located in intergenic region. Among 20 small insertions of ShT-N, most of them were on hypothetical proteins (n = 2), pseudogenes (n = 5), or intergenic regions (n = 10) (Supplemental Table 2).

### Existence of mycolactone biosynthesis proteins produced by a giant plasmid

During the course of our experiments we noticed that the avirulent MU-8 strain was also non-pigmented. Although this strain carries the giant plasmid, several deletions (see below) caused some open reading frames (ORFs) to become dysfunctional^12,13^. Therefore, we hypothesized that non-pigmented ShT-N would be similar to MU-8 in that it does not produce functional mycolactone. To test this hypothesis, we first isolated mycolactone from culture filtrates by thin-layer chromatography (TLC). Mycolactone was not detected in the ShT-N and MU-8 samples, whereas a control lipid, phthiocerol, was present. In contrast, both mycolactone and phthiocerol were present in culture filtrates from ShT-P, MU-1615, and MU-4, which are all virulent strains (Fig. 2A). In detail, we analyzed the precise structures of mycolactone from *M. shinshuense* ShT-P by electrospray ionization mass spectrometry (ESI/MS) and MS/MS^14^. The ESI/MS spectrum of acetone soluble lipids (ASLs) from the strain ShT-P detected the peaks of m/z 765.4, 763.4, 779.4 as [M + Na]^+^. In each MS/MS spectrum, the distinct ions were found m/z 359 and 429 for mycolactone A/B; m/z 357, 429 and 705 for mycolactone S1; m/z 373, 429 and 705 for mycolactone S2, respectively (Fig. 2B). The strain ShT-P produces mycolactone A/B, S1, and S2 as previously described^15^.

**Figure 3:**
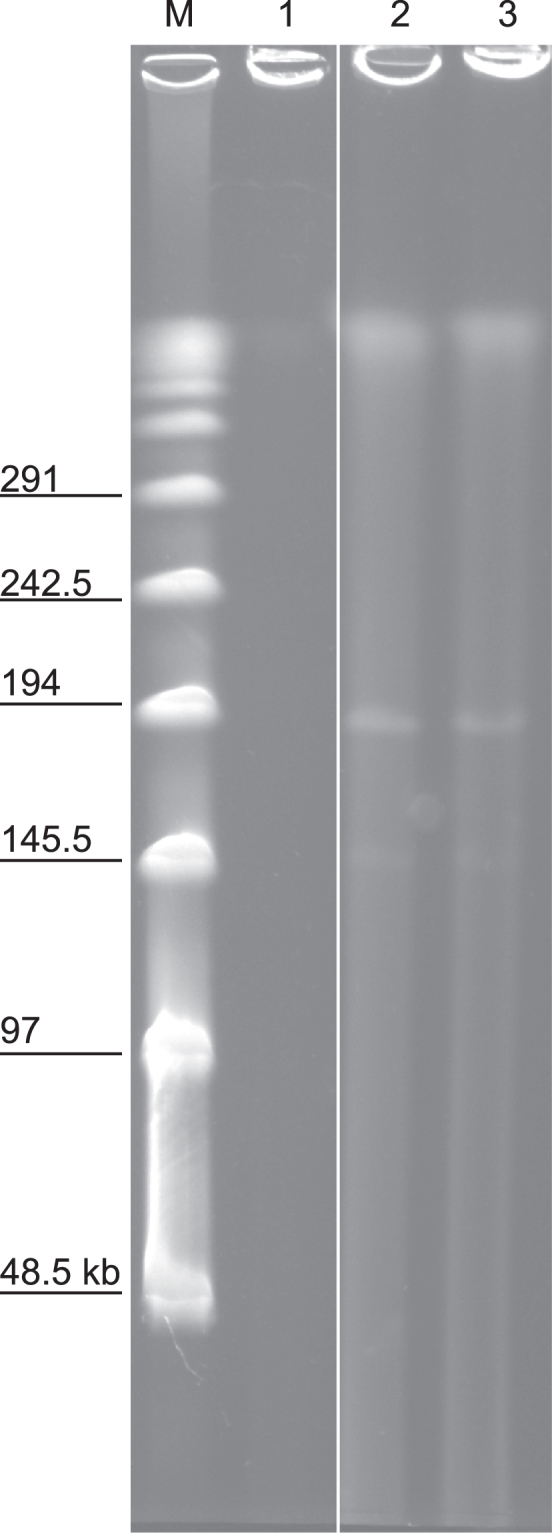
Pulse field gel electrophoresis of “*M. shinshuense*” or *M. ulcerans*. Samples were loaded as follows: lane 1, ShT-N (ATCC 33728, non-pigmented colony); lane 2, ShT-P (ATCC 33728, pigmented colony); lane 3, Sh-753 (“*M. shinshuense”*, virulent); M, lambda PFG DNA size ladder. Arrow indicates the size of a giant plasmid, approximately 167 kbps.

We confirmed the deletion of the giant plasmid in ShT-N from ShT-P by three methods: PCR, pulse field gel electrophoresis (PFGE), and WGS. We used regular PCR to check for the presence of eight ORFs that are associated with mycolactone expression and are expected to be in the giant plasmid (Table 2 and Supplemental Fig. 1)^9,16^. The ShT-P isolate produced all expected products, as did the virulent reference strains MU-4 and MU-1615. We did not detect any of the ORFs in ShT-N samples, and in the avirulent MU-8 strain only the MUP011, MUP038, and *mls*AT(II) ORFs were absent. In PFGE experiments, we detected a <194 kbp band corresponding to the size of the giant plasmid (estimated ~167 kbp^8^) in the ShT-P isolate; however, the ShT-N isolate did not produce this band (Fig. 3 and Supplemental Fig. 2)^17^. Results for WGS analysis were consistent with the previous experiments in that sequences from the giant plasmid were absent from the ShT-N isolate, but were present in the ShT-P isolate (data not shown).

**Table 2:** PCR detection of eight giant plasmid-associated genes in *M. ulcerans* or “*M. shinshuense”* strains.

	pMUM001 marker genes							
	*repA*	*parA*	MUP011 (STPK)	*mls*	*mls*AT	MUP038 (TEII)	MUP045 (KSIII)	MUP053 (p450)
ShT-P	+	+	−	+	+	+	+	+
ShT-N	−	−	−	−	−	−	−	−
Sh-753	+	+	−	+	+	+	+	+
MU-4	+	+	+	+	+	+	+	+
MU-1615	+	+	+	+	+	+	+	+
MU-8	+	+	−	+	−	−	+	+

ShT-P: ATCC 33728, pigmented strain; ShT-N: ATCC 33728, non-pigmented strain; Sh-753: “*M. shinshuense”*, virulent; MU-4: *M. ulcerans* 97–107 African strain, virulent; MU-1615: *M. ulcerans* Malaysian strain, virulent; MU-8: *M. ulcerans* 5143 Mexican strain, avirulent; STPK, serine/threonine protein kinase gene; *mls*AT, acyltransferase domain of *mls*; TEII, type II thioesterase gene; KSIII, type III ketosynthase gene; p450, p450 hydroxylase gene.

### Cytotoxicity of “*M. shinshuense*” ShT-P and ShT-N isolates

Previous work demonstrated that mycolactone can induce apoptosis and eventually cell death in various primary cells or cell lines, including murine L929 fibroblast cells^10,18^. To evaluate the cytotoxicity of the ShT-P and ShT-N strains, we incubated L929 cells for 24 hours in the presence of filter-sterile bacterial culture supernatant (SF). L929 cells rounded up and detached from the bottom of the culture dish when exposed to ShT-P SF, Sh-753 SF, or MU-4 SF, suggesting that mycolactone induced cellular damage. In contrast, cells retained a normal morphology when incubated with ShT-N SF or MU-8 SF (Supplemental Fig. 3).

### Lethality of “*M. shinshuense*” ShT-P and ShT-N isolates in mice

**Figure 4:**
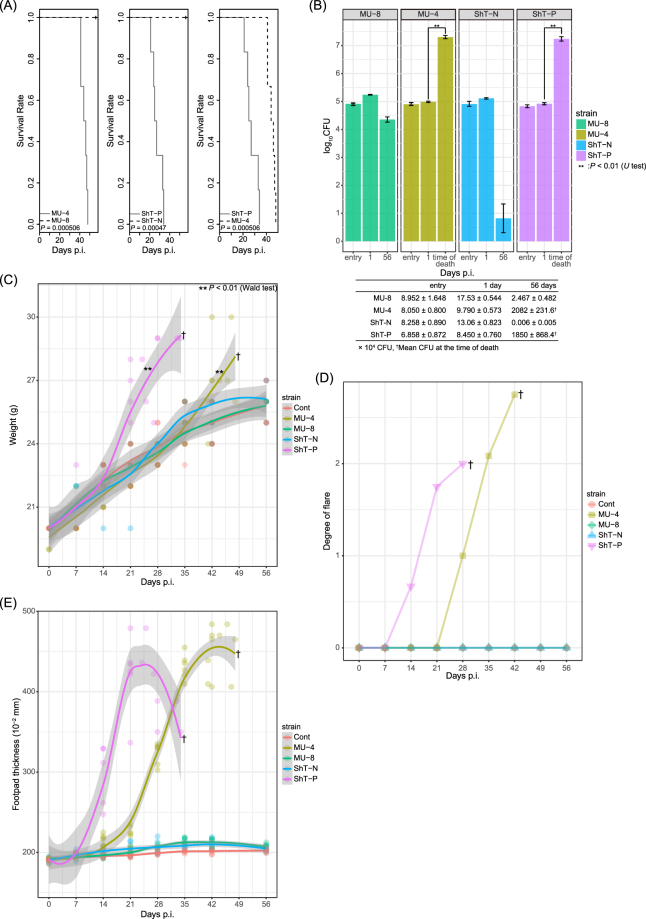
(**A**) Survival curve of infected mice after inoculation with whole, live bacteria. Similar bacterial loads of “*M. shinshuense”* (ShT-P or ShT-N) or *M. ulcerans* (MU-4 or MU-8) were inoculated into the footpads of BALB/c mice (n = 6, each group). The mice inoculated with ShT-P died within 5 weeks of inoculation (middle, right panels) whereas those inoculated with ShT-N were all alive even after 8 weeks (middle panel). **(B)** Bacterial load in hind footpads of mice inoculated with “*M. shinshuense*” or *M. ulcerans* at entry, 1 day and 8 weeks after inoculation or at the time of death. Bacterial load is shown as log_10_ CFU/foot pad. ShT-N CFUs were absent after 8 weeks. **(C)** Body weight of infected mice or control mice after inoculation with bacteria. Body weight of infected ShT-P and MU-4 was significantly increased relative to that of control animals (p < 0.01). **(D)** Degree of erythema in the hind footpads of the inoculated mice. 1+ : slight, 2+ : moderate, 3+ : severe. **(E)** Measurement of hind footpad swelling induced by “*M. shinshuense”* or *M. ulcerans*.

Several reports showed that *M. ulcerans* mycolactone causes apoptosis *in vitro* and/or ulcers *in vivo*^12,18–21^. To verify that the lack of mycolactone can prevent disease *in vivo*, we inoculated live bacilli from ShT-P or ShT-N isolates into the footpads of BALB/c mice. All mice given ShT-P were dead within 5 weeks of inoculation, which was significantly faster than those given MU-4, a control virulent strain (p < 0.00051, Fig. 4A right panel). However, all mice inoculated with ShT-N or MU-8 survived for the full course of the experiment (>8 weeks). The number of inoculated bacteria and the infectivity measured as colony-forming units (CFU), 24 hours after inoculation did not have a statistically significant (Fig. 4B, entry and 1 day). For mice given ShT-P, local swelling and erythematous lesions and/or ulcerative and necrotic lesions were observed. Two weeks after the inoculation, these mice showed weight, erythema and swelling of the hind footpads that increased over time **(**Fig. 4C–E), whereas necrotic skin lesions were observed after three weeks (data not shown). At the time of death or 8 weeks post-inoculation, significant swelling and/or erythema were observed in mice inoculated with ShT-P, but not ShT-N (Supplemental Fig. 4).

### Histology and bacterial burden of local lesions

**Figure 5:**
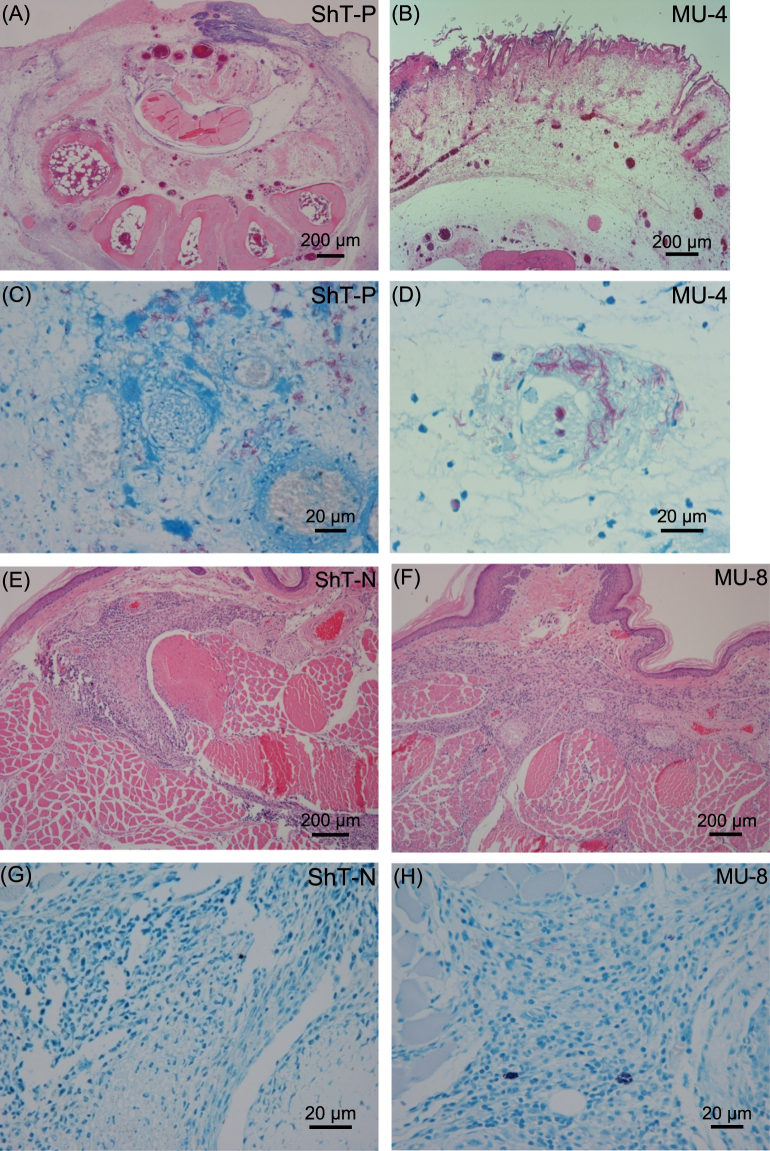
Representative images of skin lesion tissue sections stained with H&E (**A,B,E,F**) or Fite-Faraco (**C,D,G,H**) at the time of death (ShT-P and MU-4) or 8 weeks after inoculation (ShT-N and MU-8). *M. ulcerans* MU-4 and MU-8 were used as pathogenic positive and negative controls, respectively.

At the time of death, mice inoculated with ShT-P or MU-4 displayed remarkable, deep skin ulcers and extensive subcutaneous edema that was associated with prominent vascular dilatation and occasional thickening of the vascular wall (Fig. 5A). The dermis was edematous with little inflammatory infiltration and mild fibrinous exudation (Fig. 5B). Large numbers of acid-fast bacilli formed clusters mainly in the edematous stroma and focally in the monocytes (Fig. 5C,D). In control mice, some cell infiltration and mild granulomatous change occurred at the inoculation site, but neither necrotic change nor ulceration was seen (Fig. 5E–H). The number of bacilli purified from the hind footpads was significantly higher in ShT-P- or MU-4-inoculated mice compared to ShT-N- or MU-8 inoculated mice (Fig. 4B).

## Discussion

During routine culture, we isolated a mycolactone-deficient strain of *M. ulcerans* subsp. *shinshuense* from a mycolactone-producing reference strain and confirmed the toxicity of mycolactone. The ORFs required for synthesis of this polyketide toxin (*mls*) are carried on a giant plasmid found in many strains of *M. ulcerans*^22^. We showed that ShT-N lacks this giant plasmid by several ways including WGS analysis and that this plasmid is essential for *M. ulcerans* and “*M. shinshuense”* pathogenicity *in vitro* and *in vivo*.

Differential diagnosis among nontuberculosis mycobacteria is important since pathogenesis and drug susceptibility can differ significantly among similar species or even subspecies^23^. Genetic analysis techniques, such as MLST, and conventional biochemical analyses are routinely performed to obtain a differential diagnosis^23,24^. More recently, WGS has been used to compare species and/or subspecies^23^. In this study, we demonstrated that WGS analysis can be used to directly compare differences in nucleotides between the ShT-P genome and that of ShT-N by analyzing large numbers of nucleotides to show that the genomes of these two strains were almost identical except for non-synonymous alterations in three ORFs and for frameshifts in five ORFs. These SNPs or Indels could be used in the future to induce deletion of the giant plasmid from *“M. shinshuense”* strains or reduce *“M. shinshuense”* pathogenicity. Especially, recent several studies suggest that GntR family transcriptional regulator might be associated with bacterial virulence or pathogenicity^25–27^.

Mycolactone is a powerful toxin in mammals^11^. In a previous study, subcutaneous injection of purified toxin into guinea pigs produced severe skin lesions that are similar to human ulcers^12^. Moreover, when *M. ulcerans* MU-4 was inoculated in the footpads of mice, necrotic changes without apparent inflammatory infiltration were observed and the affected mice died^21^. Our study showed that loss of the giant plasmid and mycolactone production from “*M. shinshuense”* led to a complete loss of bacterial pathogenicity and completely prevented death of inoculated mice. This result supports the idea that mycolactone is critical for virulence of *M. ulcerans* strains.

Heterogeneity of mycolactone production by various clinical isolates of *M. ulcerans* has been reported by others^13,16,22,28,29^. For the MU-8 strain, the giant plasmid is present, but its lack of several key elements for mycolactone synthesis inhibits production of a functional toxin (Fig. 2A). However, studies that compared mycolactone-producing isolates to non-producing isolates from different sources^13^ were unclear on how much change in virulence occurred. We observed a color change of bacterial colonies during culture of the original giant-plasmid-harboring strain of “*M. shinshuense”* on Ogawa egg medium slants. We also noted that *mls*AT(II) and/or MUP038 might be critical for virulence because both avirulent strains ShT-N and MU-8 were deficient in these genes. On the other hand, the absence of MUP011 from the virulent strains ShT-P and Sh-753 suggests that this gene might not be critical for pathogenesis.

A comparison of the survival curves for mice given ShT-P (*“M. shinshuense”*) and MU-4 (*M. ulcerans*) indicated that the pathogenesis of *“M. shinshuense”* in mice might be stronger than that of *M. ulcerans* (Fig. 4A). The described below is several speculations to explain the difference. (1) Because both strains possess mycolactone, this difference may be due chromosomal changes. We are currently performing genomic comparison analyses between *“M. shinshuense”* and *M. ulcerans* to explore this possibility. (2) Besides with the difference of chromosome, we found the quantitiy of containing plasmids were different, roughly four plasmids harbored in one chromosome in ShT-P, whereas approximately two plasmids in one chromosome in Agy99, typical strain of *M. ulcerans*^30^. (3) The quality of mycolactone might be different. *M. ulcerans* pocesss mycolactone A/B whereas “*M. shinshuense*” mycolactone A/B, S1, and S2. (4) The *in vivo* growth rate of bacteria might be different, which may lead the difference of mycolactone production.

We observed that the number of CFU increased between day1 and time of death for mycolactone producing mycobacteria (ShT-P and MU-4) and not for mycolactone deficient mycobacteria (ShT-N and MU-8) (Fig. 4B). This result suggests that the persistence of mycobacteria in mice tissue is due to the presence of mycolactone. In addition, the clearance of CFU is complete for strain ShT-N but not for the non-mycolactone-producing strain MU-8 (Fig. 4B). As neither strains produce functional mycolactone, ShT-N lost a plasmid but MU-8 still has a mutated plasmid, which may lead the difference of the clearance.

In conclusion, we made an avirulent mycolactone-deletion mutant strain directly from the virulent prototypic strain. Our assessment of this strain indicates that “*M. shinshuense”* pathogenicity was mainly due to mycolactone production. Surprisingly, “*M. shinshuense*” pathogenesis was stronger than that of *M. ulcerans*. Because the genomes of these two strains are nearly identical and the most significant differences are whether the giant plasmid is present, this pair would be beneficial for future pathogenicity comparison analyses of the *M. ulcerans* group.

## Materials and Methods

### Bacterial strains

The first clinical isolate of “*M. shinshuense*” was deposited in the ATCC (ATCC 33728) and is considered as the reference strain^9^. All bacteria were cultured as previously described^9^. We isolated ShT-P (pigmented) and ShT-N (non-pigmented) strains from “*M. shinshuense*” strain ATCC 33728s, which was received in December 2004 from The Research Institute of Tuberculosis, Japan (Fig. 1). The virulent “*M. shinshuense”* clinical isolate JATA 753 (Sh-753)^31^, virulent *M. ulcerans* 1615 Malaysian strain (MU-1615), virulent *M. ulcerans* 97–107 African strain (MU-4), and avirulent *M. ulcerans* 5143 Mexican strain (MU-8) were used in all experiments. MU-1615, MU-4, and MU-8 were generously provided by Dr. Françoise Portaels (Antwerp, Belgium)^9,13^. *M. marinum* ATCC 927^T^ and *M. pseudoshottsii* JCM 15466^T^, provided from the Riken Japan Collection of Microorganisms, were used for comparison in some genomic assays, such as MLST^6^.

### Examination of phenotypic properties

Bacterial morphology and acid–alcohol fastness were determined by Ziehl-Neelsen staining as previously described^32^. Colony morphology, pigmentation, and the ability to grow at various temperatures (25 °C, 32 °C, 37 °C, and 42 °C) were observed on Middlebrook 7H11-OADC agar (Nippon Becton Dickinson, Tokyo, Japan) and 2% Ogawa egg medium slants (Kyokuto Pharmaceutical Industrial, Tokyo, Japan). The following biochemical tests were performed as previously described^6,33^: Tween 80 hydrolysis, nitrate reductase, semiquantitative catalase, heat-stable catalase (68 °C), arylsulfatase activity (3-day), urease activity, pyrazinamidase, iron uptake, and MPB64 Ag production.

### Thin-layer chromatography (TLC)

Mycolactone analyses were performed using silica gel thin-layer chromatography (TLC) as described previously with some modifications^34^. ASLs were prepared from culture filtrates and analyzed by silica TLC developing with the solvent; chloroform-methanol-water (90:10:1, vol/vol/vol). Lipids were visualized by a phosphomolybdic acid ethanol solution^13^. The presence of phthiocerol and mycolactone was determined by mass spectroscopy^15,35^. In briefly, the ASLs dissolved in ethanol were directly perfused into an electrospray ionization source on Thermo Finnigan LCQ Advantage Max LC/MS/MS ion trap mass spectrometer (Thermo Scientific) by using syringe pump YSP-101 (YMC CO. LDT.). The ESI/MS conditions were optimized to the synthetic mycolactone A/B provided by Yoshito Kishi (Harvard University)^14^. The conditions were as follows, positive mode; infusion rate, 1.0 µl/min; dry temperature, 320 °C. The mycolactones were determined by the molecular ions of ESI/MS and their characteristic fragment ions of ESI/MS/MS.

### Pulse field gel electrophoresis (PFGE)

PFGE was performed as previously described^36,37^. In brief, plugs containing undigested DNA were loaded onto 1% agarose gel and run in Tris-borate-EDTA buffer (0.025 M Tris, 0.5 mM EDTA, 0.0254 M boric acid). PFGE was carried out using a CHEF-DR II system (Bio-Rad Laboratories, Richmond, CA) at 14 °C and 200 V for 20 h. The lambda PFG ladder (Bio-Rad) was used as a molecular size marker. After staining with ethidium bromide, the gel was photographed using a UV transilluminator.

### DNA extraction, PCR, and multilocus sequence typing (MLST)

DNA was extracted as previously described^35,38–40^. In brief, a loop of bacilli was suspended in 400 μl sterilized phosphate buffered saline supplemented with 0.05% Tween 80, and then stored at −80 °C. The frozen sample was crushed with zirconia beads (2 mm diameter) in a bead beating instrument (MagNA Lyser, Roche Diagnostics). Total genomic DNA was purified from the crushed suspension using a High Pure PCR Template Preparation Kit (Roche Diagnostics) according to the manufacturer’s instructions and stored at −20 °C.

PCR products from eight regions of pMUM001 that code for mycolactone-producing enzymes (*plasmid replication protein A* (*repA*) (413 bp), *plasmid maintenance protein A* (*parA*) (501 bp), *M. ulcerans* plasmid (MUP) 011 (serine/threonine protein kinase, 479 bp), *M. ulcerans* type I polyketide synthases loading domain (*mls*(load)) (560 bp), *M. ulcerans* type I polyketide synthases acyltransferase domain (*mls*AT(II)) (504 bp), MUP038 (type II thioesterase, 500 bp), MUP045 (type III ketosynthase, 496 bp), and MUP053 (p450, 500 bp)) were compared between ShT-P, ShT-N, Sh-753, MU-4, MU-1615, and MU-8^16^. The primers were previously reported and are listed in Supplemental Table 3^16^.

The sequences of the 16S rRNA, the internal transcribed spacer between the 16S and 23S rRNA genes (ITS region), *rpoB*, and *hsp65* genes were analyzed with the primers listed in Supplemental Table 1^6,41^. Amplified PCR products (sizes shown in Supplemental Table 1) were directly sequenced using the ABI Prism 310 PCR genetic analyzer (Applied Biosystems, Foster City, CA)^9,35,38–40^. ShT-P and ShT-N strains were compared to six related reference strains: “*M. shinshuense”* Sh-753, *M. ulcerans* MU-4, *M. ulcerans* MU-1615, *M. ulcerans* MU-8^10^, *M. marinum* ATCC 927^T^, and *Mycobacterium pseudoshottsii* JCM 15466^T^. A similarity search was also performed with ShT-P, ShT-N, and other mycobacterial reference strains using the DNA Data Bank of Japan (DDBJ)^6^.

### Short-read DNA sequencing

WGS analysis of the ShT-P strain is described elsewhere^8^. The DDBJ/ENA/GenBank accession numbers of the ShT-P chromosome and giant plasmid are AP017624 and AP017625, respectively, whereas that for the ShT-N chromosome is AP017635. The draft genome sequence of the ShT-N strain was determined using the 454 GS FLX Titanium system (Roche Diagnostics). A total of 469,267 single-end and 109,625 paired-end (8 kb insert) reads were assembled with the GS Assembler software version 2.6 (Roche) into one scaffold containing 249 gaps. Illumina paired-end reads (150 × 2; 1,369,886 reads) obtained by a MiSeq sequencer (Illumina, San Diego, USA) were used for sequence-error correction^8,42–46^.

### SNP calling

To detect SNPs between ShT-P and ShT-N strain, we performed the best practice method presented by the authors of the Genome Analysis Toolkit (GATK)^47^. Briefly, raw sequence reads (FASTQ format) of ShT-N were mapped against the genome sequence of ShT-P, and SNPs and insertions/deletions (indels) were detected from the resulting mapping. Then, the BWA program^48^. was used to map reads against the genome of ShT-P, resulting in a file of aligned reads in sequence alignment/map (SAM) format^49^. Using utilities in the SAM tools package^49^, the SAM file was converted into the binary alignment/map (BAM) format for subsequent processing. Duplicate reads were detected and marked using the “MarkDuplicates” feature of Picard (http://broadinstitute.github.io/picard/). The “RealignerTargetCreator” and “IndelRealigner” functions of GATK^50,51^ were used to improve the alignment of the reads. The GATK function “HaplotypeCaller” was used to generate a variant call format (VCF) file^52^, which counts detected SNPs and indels. The “VariantFiltration”, “BaseRecalibrator” and “PrintReads” functions of GATK were used to remove false positives by calibrating the base-quality scores. The Integrative Genomics Viewer (IGV)^53,54^ was used to visualize the detected SNPs and indels.

### Cytotoxicity assay

An assay to determine the toxicity of the different bacterial strains to a murine cell line was performed as previously described^12,18,20^. Briefly, murine L929 cells were plated at 5 × 10^4^/well in 24-well plates, and sterile filtrate from mycobacterial cultures was added to the wells. At 24 hours post-treatment, the L929 cells were inspected microscopically for cell rounding and detachment.

### *In vivo* infection of mouse footpads

The culture of bacteria in 7H9 broth was allowed to sit for 20 min for the large bacterial clumps to settle down. The resident bacterial suspension was diluted with 7H9 and its optical density was adjusted at 540 nm and then used as inoculum for infection. A suspension of PBS (25 μL) containing various loads of “*M. shinshuense*” (ShT-P or ShT-N) or *M. ulcerans* (MU-4 or MU-8) was inoculated into the footpads of 5-week-old female BALB/c mice (n = 6, each group) as previously described^21,55^. Bacterial load was measured at 24 hours and 8 weeks after inoculation or at the time of death. Hind footpads of mice were disinfected and homogenized in PBS (−) by using a glass homogenizer. The resultant homogenate was subjected to serial 10-fold dilutions with normal saline and 0.1 mL was inoculated onto 7H11 medium. The number of CFU was counted 8 weeks of incubation at 32 °C. Care and treatment of animals followed the regulations of the Animal Care and Use Committee of the National Institute of Infectious Diseases, Japan.

### Histopathological examination

Histopathological examination was performed as previously described^19^ 8 weeks after inoculation or at the time of death. After induction of deep anesthesia, mice were fixed by perfusion with 10% formalin. Hind limbs and general organs were then embedded in paraffin, cut into 4 μm sections, and histopathological examination was performed after hematoxylin and eosin (H&E) and Fite-Faraco acid-fast staining. Ulcerative lesion(s), granuloma formation(s), and acid-fast bacilli were evaluated.

### Statistical analysis

All statistical analyses were performed with R software (www.r-project.org). The survival curves were plotted according to the Kaplan-Meier method, and statistical significance between infected strains in the assay was assessed by log-lank test. To statistically assess CFUs at each time point, the Mann-Whitney *U* test was used. To statistically assess the weight of the infected mice, a GLM, which is an extension of the normal linear model, was used. The ANCOVA model, a blend of ANOVA and regression in a multiple linear model, was applied to test the effect of infection on the weight of the mice in the manner of the GLM. The statistical model was defined as *η*(*w*) = *β*_0_ + *β*_1_*t* · *s*, where *η* is the link function, w is a response variable (weight of mice), *t* is the time post-infection, *s* is the infected strains, *β*_0_ is the intercept, *β*_1_ is the fixed effect of interaction between time post-infection and infected strains, of which the explanatory variable *t* · *s* of the interaction term was defined as a product of *t* and *s* in the linear model. The *P* values calculated by the Wald test for *β*_1_ of the maximum likelihood estimation in each infected strain was used to assess whether the strain affected mouse weight.

## Electronic supplementary material

Supplemental Information
