# Supplementary material for: Naturally occurring a loss of a giant plasmid from Mycobacterium ulcerans subsp. shinshuense makes it non-pathogenic
Source: Sci Rep. 2018 May 29;8:8218. doi: 10.1038/s41598-018-26425-1 (PMC5974349; doi:10.1038/s41598-018-26425-1)
Supplement: Supplementary file 1 — Supplemental Information [file 41598_2018_26425_MOESM1_ESM.docx]

Supplemental information of Naturally occurring a loss of a giant plasmid from *Mycobacterium ulcerans* subsp. *shinshuense* makes it non-pathogenic

Kazue Nakanaga^1#^, Yoshitoshi Ogura^3,#^, Atsushi Toyoda^4^, Mitsunori Yoshida^1^, Hanako Fukano^1^, Nagatoshi Fujiwara^5^, Yuji Miyamoto^1^, Noboru Nakata^1,2^, Yuko Kazumi^2,6^, Shinji Maeda^6,9^, Tadasuke Ooka^7^, Masamichi Goto^8^, Kazunari Tanigawa^1,10^, Satoshi Mitarai^6^, Koichi Suzuki^1,11^, Norihisa Ishii^1^, Manabu Ato^1^, Tetsuya Hayashi^3^, Yoshihiko Hoshino^1^*

^1^Department of Mycobacteriology, Leprosy Research Center, ^2^Antimicrobial Resistance Research Center, National Institute of Infectious Diseases, Japan, ^3^Department of Bacteriology, Faculty of Medicine, Kyushu University, ^4^Center for Information Biology, National Institute of Genetics, Japan, ^5^Department of Food and Nutrition, Faculty of Contemporary Human Life Science, Tezukayama University, ^6^The Research Institute of Tuberculosis, Japan Anti-Tuberculosis Association, ^7^Department of Microbiology, Graduate School of Medical and Dental Sciences, Kagoshima University, and ^8^National Sanatorium Hoshizuka-Keiaien.

^#^ K. N. and Y. O. equally contributed to this work.

^9^Present affiliation: School of Pharmacy, Hokkaido Pharmaceutical University

^10^Present affiliation: Faculty of Pharma-Sciences, Teikyo University

^11^Present affiliation: Department of Clinical Laboratory Science, Faculty of Medical Technology, Teikyo University

**Supplemental Figure legends**

**
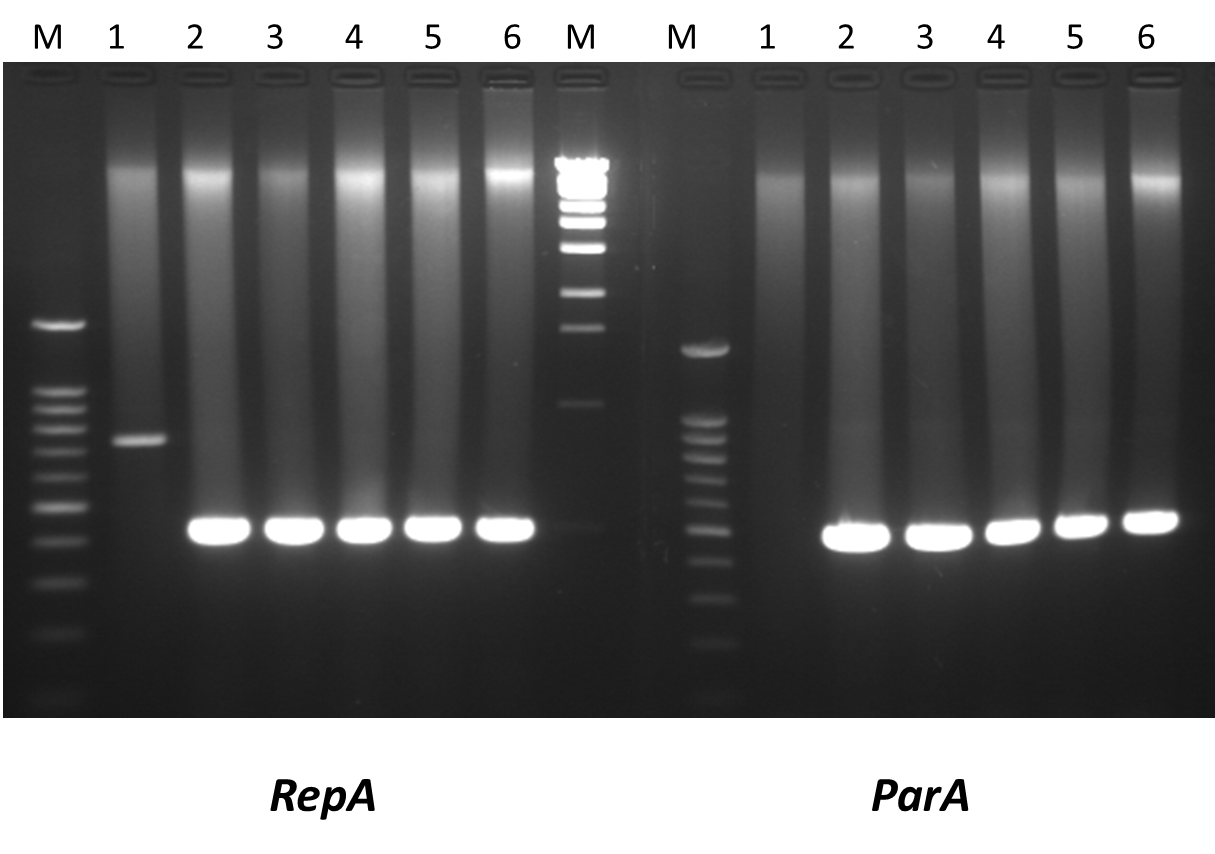
**

**Supplemental Figure 1** (Left panel) PCR products targeting to *repA* gene in pMUM001. (labelled as RepA). (Right panel) PCR products targeting to *parA* gene in pMUM001 (labelled as ParA). Strains are represented in lanes as follows: lane 1, ShT-N (ATCC33728, ivory colony); lane 2, ShT-P (ATCC33728, yellow colony); lane 3, Sh-753 (“*M. shinshuense”* JATA 753, virulent); lane 4, MU-4 (*M. ulcerans* 97-107 African strain, virulent); lane 5, MU-1615 (*M. ulcerans* Malaysian strain, virulent); lane 6, MU-8; (*M. ulcerans* 5143 Mexican strain, avilurent). M, DNA marker.


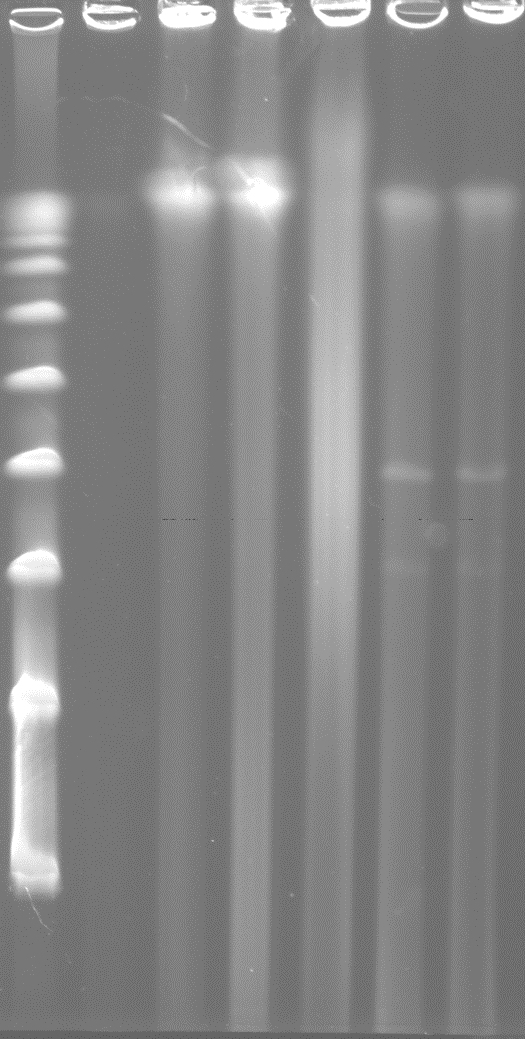


**Supplemental Figure 2** Original gel blots of Pulse field gel electrophoresis of “*M. shinshuense*” or *M. ulcerans*. Samples were loaded as follows: lanes 1-4, ShT-N (ATCC 33728, non-pigmented colony) with variety of inputs; lane 5, ShT-P (ATCC 33728, pigmented colony); lane 6, Sh-753 (“*M. shinshuense”*, virulent); M, lambda PFG DNA size ladder.

**
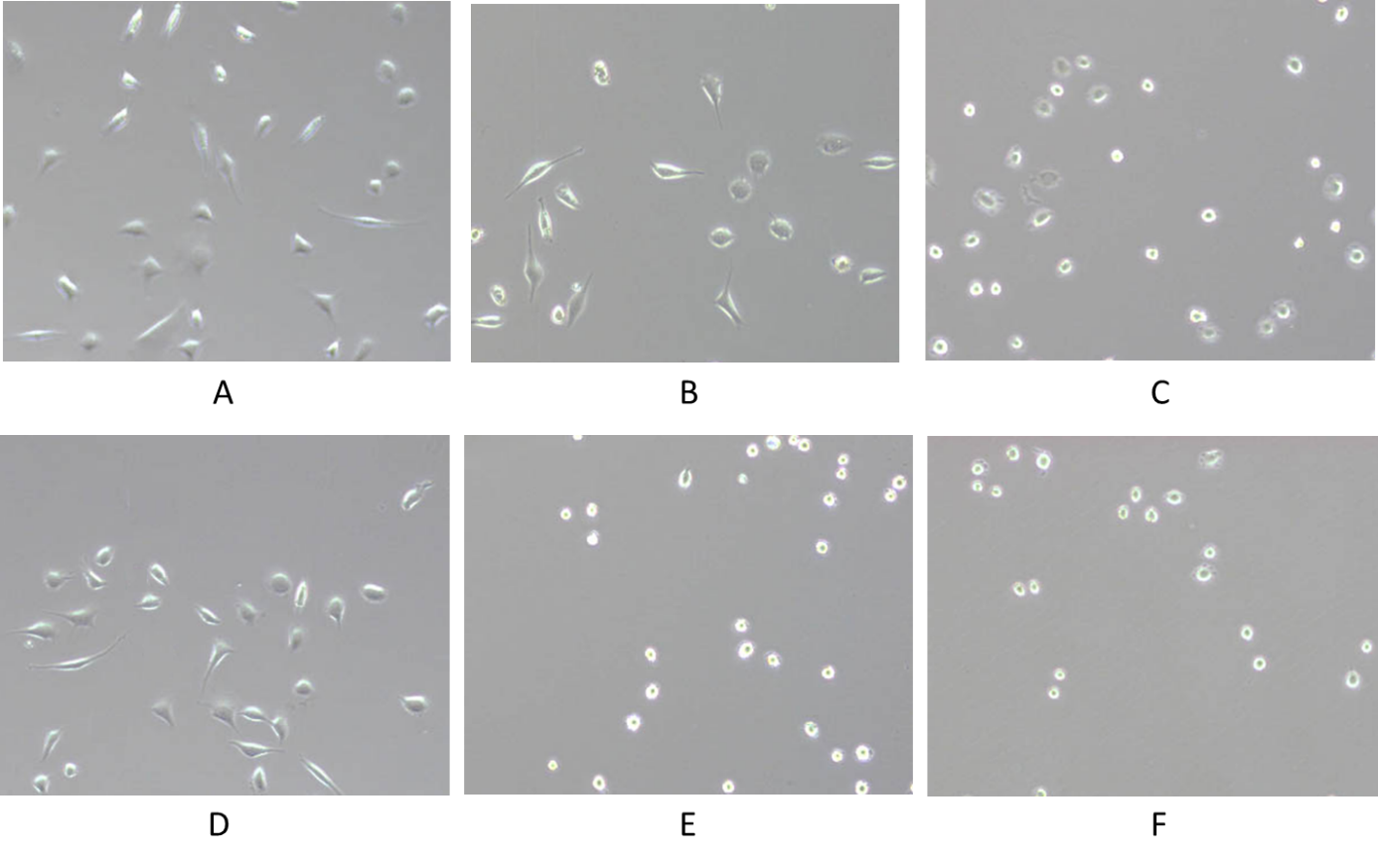
**

**Supplemental Figure 3** Effect of toxin on L929 cells. L929 cells were exposed to either 7H9 medium or sterile filtrate (SF). **(A)** L929 cells exposed to 7H9 medium for 24 h show normal structure. **(B)** L929 cells exposed to ShT-N SF for 24 h show normal structure. **(C)** L929 cells exposed to ShT-P SF for 24 h show damaged circular structure. **(D)** L929 cells exposed to MU-8 (*M. ulcerans* avirulent strain) SF for 24 h show normal structure. **(E)** L929 cells exposed to Sh-753 (“*M. shinshuense”* virulent strain) SF for 24 h show damaged circular structure. **(F)** L929 cells exposed to MU-4 (*M. ulcerans* virulent strain) SF for 24 h show damaged circular structure.

**
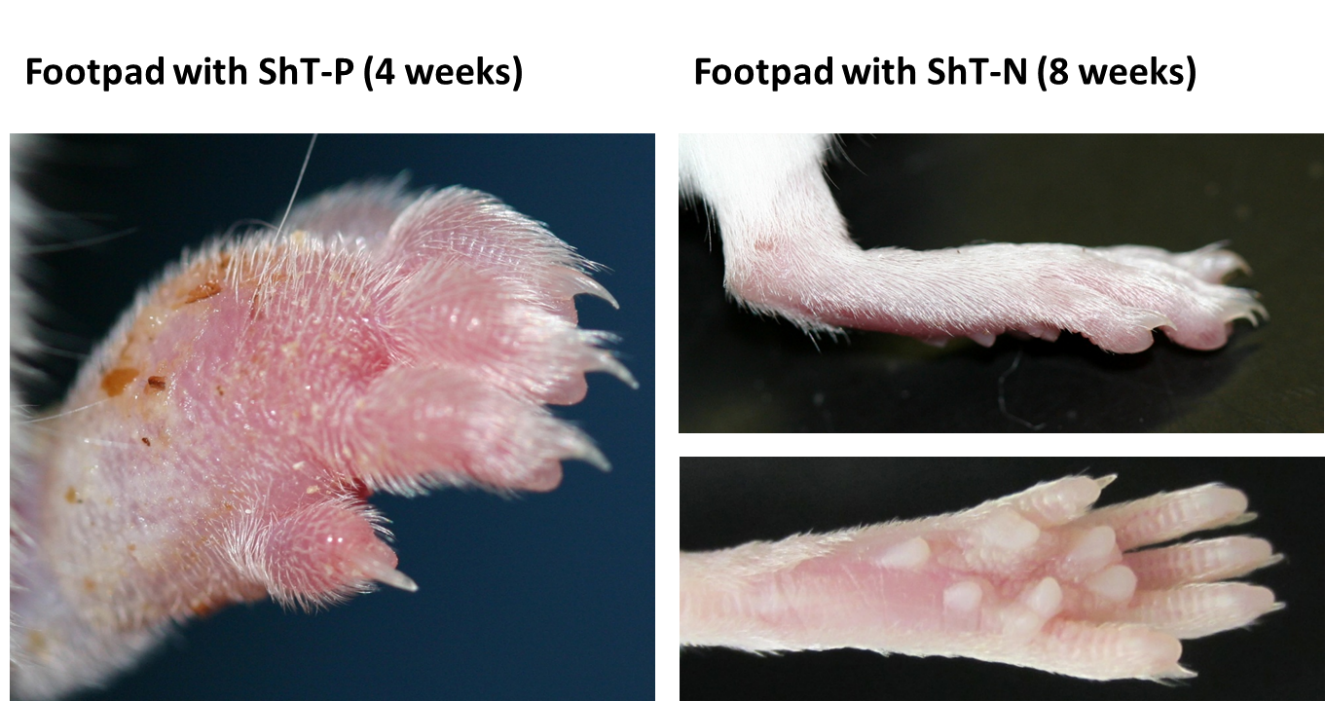
**

**Supplemental Figure 4** The left panel shows massive dermal swelling at the time of death (four weeks) in a mouse footpad inoculated with ShT-P. In the right panel, a mouse footpad lacks signs of swelling or significant ulcer formation 8 weeks after ShT-N inoculation.

Supplemental Table 1 Comparison of 16S rRNA gene sequences of M. shinshuense strains and related mycobacterial strains

|  | Nucleotides at the following *E. Coli* 16S rRNA gene sequence positions | | | | | | | | |
| --- | --- | --- | --- | --- | --- | --- | --- | --- | --- |
|  | 95 | 487-488 | 492 | 969 | 1007 | 1215 | 1247 | 1288 | 1449-1451 |
| ShT-P | T | GG | G | A | G | T | G | G | - - - |
| ShT-N | T | GG | G | A | G | T | G | G | - - - |
| Sh-753 | T | GG | G | A | G | T | G | G | - - - |
| MU-4 | T | GG | A | A | G | T | G | C | TTT |
| MU-1615 | T | GG | A | A | G | T | G | C | - - - |
| MU-8 | T | GG | A | A | G | T | G | A | - - - |
| ATCC 927^T^ | T | GG | A | A | G | T | G | A | - - - |
| JCM 15466^T^ | C | GA | A | G | T | C | A | A | - - - |

ShT-P; ATCC 33728, pigmented clone, ShT-N; ATCC33728, non-pigmented clone, Sh-753; “*M. shinshuense”* JATA753, virulent, MU-4; *M. ulcerans* 97-107 African strain, virulent, MU-1615; *M. ulcerans* Malaysian strain 1615, virulent, MU-8; *M. ulcerans* 5143 Mexican strain, avilurent., ATCC 927^T^; *M. marinum* type strain, JCM 15466^T^; *M. pseudoshottsii* type strain.

Supplemental Table 2. Comparison of SNPs and Indels between ShT-P and ShT-N.

1. SNPs

| - SNP_posiotion (as position # of ShT-P) - Locus_tag | ShT-P  by WGS | ShT-N  By WGS | protein name | Note |
| --- | --- | --- | --- | --- |
| 321417  SHTP_0294 | A | G | hypothetical protein | depth = 44 |
| 2560633  SHTP_2218 | T | C | Transposase for IS*2404* | depth = 6 |
| 2682220  SHTP_2312 | G | A | 2-hydroxyhepta-2,4-diene-1,7-dioate isomerase | non-synonymous mutation, depth = 35^a^ |
| 3017900  SHTP_2607 | C | A | MSMEG_4193 family putative phosphomutase | non-synonymous mutation, depth = 37^a^ |
| 4565747 | C | A | intergenic region | depth = 51 ^a^ |
| 4901475  SHTP_4222 | T | C | Transposase for IS*2404* | depth = 6 |
| 5005905  SHTP_4315 | A | C | GntR family transcriptional regulator | non-synonymous mutation, depth = 38^a^ |
| 5687622  SHTP_4880 | T | C | PE-PGRS family protein | depth = 22 |

^a^: The SNPs were validated by Sanger method.

1. Indels

| - Indels_posiotion   (as position # of ShT-P)   - Locus_tag | ShT-P  by WGS | ShT-N  by WGS | protein name | note |
| --- | --- | --- | --- | --- |
| 316509 | A | AC | Intergenic region | depth = 25 |
| 423403  SHTP_0392 | C | CG | PE-PGRS family protein | pseudogene, depth = 28 |
| 551997  SHTP_0504 | T | TC | cytochrome p450 | pseudogene, depth = 10 |
| 846629  SHTP_0793 | C | CG | bioF2_1, 8-amino-7-oxononanoate synthase | frameshift,  depth = 49 |
| 1301749  SHTP_1177 | A | AC | Fatty acyl-AMP ligase FadD28 and polyketide synthase | pseudogene,  depth = 22 |
| 1637574 | G | GC | intergenic region | depth = 28 |
| 1928169 | T | TG | intergenic region | depth = 21 |
| 2405481  SHTP_2101 | A | AG | *potE*, amino acid transporter | frameshift,  depth = 36 |
| 2595480 | C | CG | intergenic region | depth = 27 |
| 2746910 | T | TG | intergenic region | depth = 39 |
| 3098503 | A | AG | intergenic region | depth = 28 |
| 3420924  SHTP_2946 | A | AC | PE family protein | frameshift,  depth = 33 |
| 3668187 | C | GCC | intergenic region | depth = 12 |
| 3907346  SHTP_3387 | T | TG | hypothetical protein | frameshift,  depth = 22 |
| 4125907 | G | GC | intergenic region | depth = 22 |
| 4851147  SHTP_4185 | A | AC | hypothetical protein | frameshift,  depth = 38 |
| 4861211 | C | CG | intergenic region | depth = 11 |
| 4942649  SHTP_4256 | A | AG | amidohydrolase | pseudogene, depth = 28 |
| 5213543 | A | ACC | intergenic region | depth = 15 |
| 5786578  SHTP_4967 | T | TG | PE-PGRS family protein | pseudogene, depth = 6 |

Supplemental Table 3 Primers used in this study

| Primer | Sequence (5’- 3’) | PCR target (fragment size) | Reference |
| --- | --- | --- | --- |
| 8F16S | AGAGTTTGATCCTGGCTCAG | 16S rRNA gene, (app. 1520 bp) | ^1^ |
| 1047R16S | TGCACACAGGCCACAAGGGA |  |  |
| 830F16S | GTGTGGGTTTCCTTCCTTGG |  |  |
| 1542R16S | AAGGAGGTGATCCAGCCGCA |  |  |
| ITSF | TTGTACACACCGCCCGTC | 16S-23S ITS region, (app. 340 bp), | ^2^ |
| ITSR | TCTCGATGCCAAGGCATCCACC |  |  |
| MF | CGACCACTTCGGCAACCG | *rpoB*, (341 bp) | ^2^ |
| MR | TCGATCGGGCACATCCGG |  |  |
| TB11 | ACCAACGATGGTGTGTCCAT | *hsp65*, (441 bp) | ^3^ |
| TB12 | CTTGTCGAACCGCATACCCT |  |  |
| RepAF | CTACGAGCTGGTCAGCAATG | *repA* in pMUM001 (413bp) | ^4^ |
| RepAR | ATCGACGCTCGCTACTTCTG |  |  |
| ParAF | GCAAGCTGGGCAATGTTTAT | *parA* in pMUM001 (501bp) | ^4^ |
| ParAR | GTCCGGTCCTTGATAGGTCA |  |  |
| MUP11F | ACCACCCAAGAGTGGAACTG | serine/threonine protein kinase in pMUM001 (479bp) | ^4^ |
| MUP11R | TGTCGTGTCGAGGTATGTGG |  |  |
| MLSloadF | GGGCAATCGTCCTCACTG | *mls*(load) in pMUM001　(560bp) | ^1^ |
| MLSloadR | CAAGGGCAGTCTTGATTAGG |  |  |
| MLSAT(II)F | AACGTTGAATCCCGTTTTTG | *mls*AT(II) in pMUM001　(504bp) | ^4^ |
| MLSAT(II)R | GCACCACAAAGGAACGTCTAA |  |  |
| TEIIF | ATTCAAACGGATGCGAACTG | type II thioesterase in pMUM001　(500bp) | ^4^ |
| TEIIR | ACATTGCTGGACAAACGACA |  |  |
| MUP045F | CAGCAAGTAACGGTGGAACA | type III ketosynthase in pMUM001 (496bp) | ^4^ |
| MUP045R | ACGTGGCCCATTTGTCTTAG |  |  |
| P450F | CCCACCTCGTCGTTAGTCAT | P450 in pMUM001 (500bp) | ^4^ |
| P450R | GTGCTCGGTGATCCAGAAGT |  |  |

references

1 Springer, B. *et al.* Isolation and characterization of a unique group of slowly growing mycobacteria: description of *Mycobacterium lentiflavum* sp. nov. *J Clin Microbiol* **34**, 1100-1107 (1996).

2 Roth, A. *et al.* Differentiation of phylogenetically related slowly growing mycobacteria based on 16S-23S rRNA gene internal transcribed spacer sequences. *J Clin Microbiol* **36**, 139-147 (1998).

3 Telenti, A. *et al.* Rapid identification of mycobacteria to the species level by polymerase chain reaction and restriction enzyme analysis. *J Clin Microbiol* **31**, 175-178 (1993).

4 Nakanaga, K. *et al.* "*Mycobacterium ulcerans* subsp. *shinshuense*" isolated from a skin ulcer lesion: identification based on 16S rRNA gene sequencing. *J Clin Microbiol* **45**, 3840-3843, doi:10.1128/JCM.01041-07 (2007).
